# A bioinformatic study revealed serotonergic neurons are involved in the etiology and therapygenetics of anxiety disorders

**DOI:** 10.1038/s41398-021-01432-5

**Published:** 2021-05-20

**Authors:** Han-Kui Liu, Si-Jie He, Jian-Guo Zhang

**Affiliations:** 1 BGI-Shenzhen, Shenzhen, China; 2 Shijiazhuang BGI Genomics Co., Ltd, Shijiazhuang, China

**Keywords:** Molecular neuroscience, Genetics

## Abstract

Genetic factors contribute to the susceptibility of anxiety disorders (ADs) and responses to associated cognitive-behavioral therapy (CBT). However, the type of brain cell affected by the related genes remains unclear. Previous studies have indicated various important brain neurons associated with psychiatric disorders, highlighting the necessity to study the cellular basis of anxiety. We assembled 37 AD-related genes and 23 CBT-related genes from recent large-scale genome-wide association studies, and then investigated their cell-type specificity in single-cell transcriptome data via an expression weighted cell type enrichment method. Additionally, to investigate the cellular differences between ADs and other psychiatric disorders, we excluded the genes associated with major depressive disorder, bipolar disorder, and neuroticism, resulting in 29 AD-specific genes. Remarkably, results indicate that serotonergic neurons are significantly associated with both AD-related and CBT-related genes, despite the two gene sets showing no overlap. These observations provide evidence that serotonergic neurons are involved in the etiology and therapygenetics of ADs. Moreover, results also showed that serotonergic neurons are associated with AD-specific genes, providing a supplementary finding that is in opposition to previous studies that found no evidence for the association between serotonergic neurons and psychiatric disorders via the same strategy. In summary, the current study found that serotonergic neurons are involved in the etiology and therapygenetics of ADs, providing insights into their genetic and cellular basis. Further, this cellular difference study may deepen our understanding of ADs and other psychiatric disorders.

## Introduction

Anxiety disorders (ADs) are the most common category of mental disorders^1^, including separation anxiety disorder, selective mutism, specific phobias, social anxiety disorder, panic disorder, agoraphobia, and generalized anxiety disorder. The global prevalence of ADs has been estimated at 7.3% (range 4.8–10.9%) from 87 studies across 44 countries^2^. ADs typically occur in childhood, adolescence, or early adulthood^3^. Previous studies have identified several risk factors that contribute to ADs, including genetic factors^4^, physical and sexual abuse^5,6^, parental separation^7^, and emotional maltreatment^8^. Among these factors, genetic risk variants significantly influence the development of ADs^9,10^. Recently, genome-wide association studies (GWAS) have identified several variants that contribute to the susceptibility of ADs^11,12^, and the response to associated cognitive-behavioral therapy (CBT)^13^. However, the brain cell types associated with these variants remain to be elucidated. It is necessary to understand the biological basis of these disease-related genes to aid in the development of appropriate therapeutics. Recent studies have revealed specific cell types in brain tissues are associated with neurological diseases^14,15^, including major depressive disorder (MDD), bipolar disorder (BIP), neuroticism (NEU), autism spectrum disorders (ASDs), schizophrenia, Alzheimer’s disease, and Parkinson’s disease. These studies not only have highlighted the involvement of brain cell types in neurological diseases, but have also provided an empirical method named expression weighted cell type enrichment (EWCE) to investigate the cellular basis of neurological disease-related genes in single-cell transcriptomes of mice brains. In the current study, we applied the same strategy^16^ to analyze the cellular basis of ADs. Results indicate that serotonergic neurons are significantly associated with AD-related genes and CBT-related genes.

## Materials and methods

### Cell-type expression specificity

We applied the EWCE R-package (https://github.com/NathanSkene/EWCE) reported by Zeisel’s study^16^ to investigate cell-type expression specificity of AD-related genes. The EWCE method was demonstrated to be a feasible approach to study the expression specificity of a gene list across several different cell types with single-cell transcriptomes^14,15^. The EWCE method employs various single-cell transcriptome datasets^17–20^ from mice brain regions, including neocortex, hippocampus, hypothalamus, striatum, and midbrain. These data were generated by an identical method in Karolinska Institutet and were observed with no important batch effects. A total of 9970 cells were merged into a matrix^14^ that annotates 24 brain cell types (e.g., pyramidal neurons, interneurons, oligodendrocytes, astrocytes, microglia, vascular endothelial cells, mural cells, and ependymal cells). Cell types were identified via a backspin algorithm described in corresponding studies associated with the dataset. The EWCE method calculates the average expression level of gene in each cell type and then calculates the specificity of gene in each cell type. The specificity is calculated by the mean expression in one cell type divided by the mean expression in all cell types. For a list of target genes, EWCE calculates the cell-type specificity of target genes and then estimates the *P*-value of specificity of target genes compared with the specificity of background genes via a bootstrap method. This bootstrap method randomly samples 10,000 gene lists with the same number of target genes from all the genes as background genes, and then estimates the distribution of specificity of background genes. *P*-values of specificity from multiple tests were adjusted by the false discovery rate (FDR) method.

### Genes related to ADs

We assembled 37 AD-related genes (Supplementary Table 1) from four recent, large-scale, genome-wide association studies^11,12,21,22^ to examine cell-type specificity. We identified significant loci by a *P*-value at the threshold <0.05/100,000 from the association analysis and significant genes by a *P*-value at the threshold <0.05/20,000 from gene-level analysis. We also searched the GWAS catalog database^23^ with the keyword “anxiety disorders” and accessed various AD-related loci from 32 studies related to ADs. However, there are three issues with these studies: 1) eight studies focused on other disorders, including MDD and ASDs; 2) the influence of genetic factors is unclear among ADs, obsessive-compulsive disorder, and NEU; 3) the loci reported by the GWAS catalog was not entirely consistent with the loci reported by the studies. Specifically, the GWAS catalog reported many more loci than the corresponding study. Because of these paradoxes, we did not use the loci reported by the GWAS catalog database in our subsequent study.

### Genes related to CBT of ADs

We assembled 23 genes that were evident in response to the CBT of ADs from a previous summary^13^ and four large-scale genome-wide association studies^24–27^ (Supplementary Table 2). Previous studies have indicated that no loci are strongly associated with treatment outcomes of ADs. However, variants that met the criteria for suggestive significance have been reported. We combined the eight genes with evidence and the 15 genes with suggestive significance as CBT-related genes.

### Genes related to MDD, BIP, and NEU

To access as many psychiatric disorder-related genes as possible, we examined loci reported by the GWAS catalog. We searched the GWAS catalog database with the keywords “major depressive disorder,” “bipolar disorder,” and “neuroticism.” We identified significant loci by the *P*-value at a threshold <0.05/100,000. Finally, we retained 855 genes (Supplementary Table 3) associated with MDD and/or BIP, and 422 genes (Supplementary Table 4) associated with NEU.

### Time-specific gene expression analysis

We investigated the time specificity of CBT-related genes and AD-related genes via an cell-type specific expression analysis (CSEA) tool^28^ available online (http://genetics.wustl.edu/jdlab/csea-tool-2/). The CSEA tool employs published RNA-sequence data from the human brain^29^. RNA-sequence data were filtered by the gene annotations in the reference sequence database to include only well-annotated protein-coding genes. Transcripts below a background of 0.3 reads per kilobase per million were excluded. Original data were aggregated into six major regional divisions across ten developmental periods. Cell-specific genes were identified by specificity index *p*-value (pSI) across four thresholds^30^ (0.05, 0.01, 0.001, 0.0001). Lower pSI values represented more specific genes. Significances were identified by FDR-adjusted *P*-values at <0.05 across all periods at the pSI threshold.

## Results

**Fig. 1:**
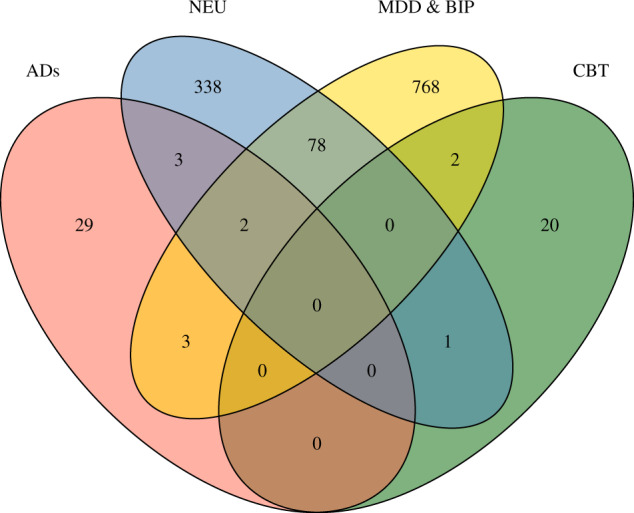
Venn diagram of the genes related to ADs, CBT, MDD & BIP, and NEU. Number in area shows the gene number in corresponding overlap. We note that ADs share few genes with MDD & BIP and NEU, and no genes with CBT.

**Fig. 2:**
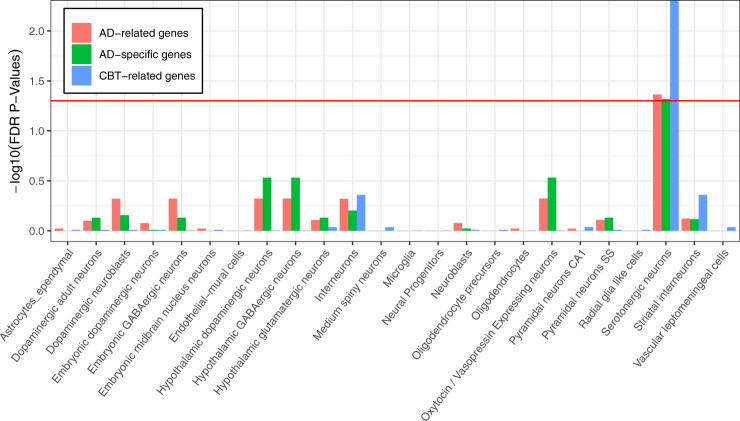
Association of brain cell types and three gene panels. The red line indicates the significance threshold (*P* < 0.05) after FDR adjustment. Histograms accessing the red line show that the serotonergic neurons are significantly associated with AD-related genes, AD-specific genes, and CBT-related genes.

We assembled 37 AD-related genes from recent large-scale genome-wide association studies and accessed the genes related to MDD, BIP, and NEU from the GWAS catalog. The overlap of the genes related to these diseases are shown in Fig. 1. Approximately 80% of AD-related genes are exclusive. This result confirmed the necessity to investigate the cell types of AD-related genes. To accomplish this goal, we employed the EWCE method developed by Skene and Grant^16^. We applied the EWCE R-package to calculate the cell-type specificity of AD-related genes in the single-cell transcriptome data of the mice brain. Remarkably, we found that the serotonergic neurons are significantly associated with AD-related genes (Fig. 2), suggesting involvement of serotonergic neurons in ADs. This observation is consistent with a previous study^31^. To avoid gene biases related to other psychiatric disorders, we excluded overlaps of genes between ADs and MDD, BIP, and NEU, and retained 29 AD-specific genes. We replicated the association between serotonergic neurons and AD-specific genes (Fig. 2). We also investigated the cell types associated with genes related to MDD, BIP, and NEU. The results exhibited no evidence for an association between serotonergic neurons and genes related to the three disorders listed above (i.e., MDD, BIP, and NEU) or the overlap of these genes and those related to ADs (Supplementary Table 5).

**Fig. 3:**
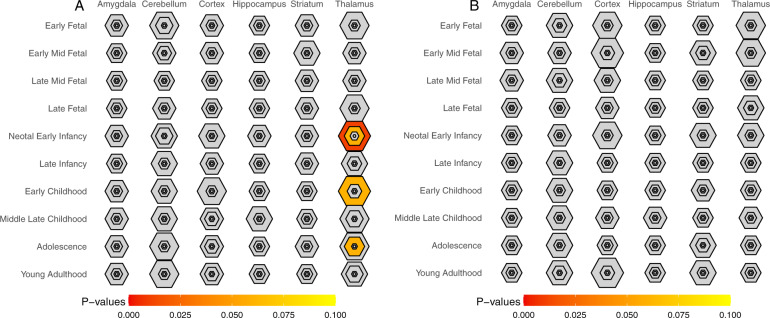
Time-specific expression of CBT-related genes (A) and AD-related genes (B). The size of the hexagons from outside to center correspond to the pSI thresholds (0.05, 0.01, 0.001, 0.0001), respectively. Colors indicate the FDR-adjusted *P*-values. The CBT-related genes show significant expression during early infancy, early childhood, and adolescence in the thalamus.

Subsequently, we explored the involvement of cell types in the treatment of ADs. CBT is the most extensively tested form of psychological therapy recommended for the treatment of anxiety and depressive disorders^32^. In the field of therapygenetics, genetic factors have been shown to affect the response to CBT of ADs^13^. However, it remains unclear when and where the associated genes are active. To identify the specific cell type associated with the psychological treatment of ADs, we assembled 23 genes related to CBT of ADs and investigated cell-type specificity via the same strategy described above. Interestingly, we found that serotonergic neurons were also significantly associated with the treatment of ADs (Fig. 2), despite CBT-related genes and ADs-related genes (Fig. 1) sharing no overlap. Additionally, we investigated the time specificity of CBT-related genes in the human brain via the CSEA tool^28^. CBT-related genes were significantly expressed during early infancy, early childhood, and adolescence (Fig. 3A). We also investigated the time specificity of AD-related genes and found the highest time-specificity during young adulthood in the cortex (*P* = 0.014). However, no significant time-specificity for AD-related genes was retained by the FDR-adjusted *P*-values at the threshold <0.05 (Fig. 3B).

## Discussion

Previous studies^14,15^ have shown that psychiatric disorders (e.g., MDD, BIP, NEU, ASDs, schizophrenia, intellectual disability, and anorexia nervosa) are predominantly associated with projecting excitatory and inhibitory neurons^14,15^. However, no evidence has indicated the involvement of serotonergic neurons in psychiatric disorders across all single-cell transcriptome datasets. NEU had the strongest signal in serotonergic neurons, but the association did not survive multiple testing correction (Supplementary Table 5). Via the same strategy and single-cell transcriptome dataset, our study showed that serotonergic neurons were associated with ADs, providing a supplementary finding for psychiatric disorders.

To our knowledge, MDD, BIP, and NEU are strongly correlated with ADs. MDD and BIP are frequently accompanied by anxiety symptoms. However, the typical features of MDD, such as anhedonia and hopelessness, are not inherent in ADs^33^. NEU has been proposed as a strong maker for predicting ADs. The genetic correlations between generalized anxiety disorder and NEU were estimated to be high in men (1), but different in women (0.58)^34^. In our study, the genes related to ADs, MDD, BIP, and NEU showed overlap. However, the specific genes related to each disorder also indicated different genetic contributions to psychiatric disorders. Underlying the psychiatric disorder-related genes, previous research has indicated MDD is associated with neuroblasts and interneurons; BIP is associated with medium spiny neurons, pyramidal neurons SS and CA1, and interneurons; and NEU is associated with pyramidal neurons SS and CA1, medium spiny neurons, neuroblasts, and adult dopaminergic neurons. Here, we showed ADs were associated with serotonergic neurons. These genetic and cellular differences may provide insight into the etiology of ADs and other psychiatric disorders.

Although we observed a different involvement of cell type in psychiatric disorders, we cannot rule out the involvement of other cell types in ADs or the involvement of serotonergic neurons in other psychiatric disorders. Our study did not investigate these possibilities. The observed association was determined by the EWCE algorithm based on a group of target genes assembled from GWAS. The EWCE algorithm was designed to focus on the main characteristics of cell-type specificity of all target genes. In these studies, the target genes may not be a comprehensive reference for a disease. It is also possible that the algorithms may not have power to detect all the characteristics of a comprehensive reference of disease-related genes. Despite the limitations in our study, we observed an association between serotonergic neurons and genes related to ADs and CBT of ADs. Moreover, we indicated that the CBT-related genes are specifically expressed during early childhood and adolescence. To our knowledge, CBT is effective for the treatment of ADs in children and adolescents^35,36^, but its active mechanism remains unclear. CBT is known to induce changes in brain activation^37^ of patients with ADs as assessed via neuroimaging. Genetic studies have provided an additional mechanism that specific gene expression is associated with the efficacy of CBT for the treatment of ADs^26^ and stress disorders^38^. The time-specific characteristic of gene expression may deepen our understanding of the biological mechanism of CBT in young individuals. Additional investigation for the time specificity of CBT-related genes in various brain cell types is needed.

Previous studies have primarily focused on the specific neurons associated with risk genes of psychiatric disorders^14,15^. Our study revealed that serotonergic neurons are associated with the treatment of ADs. The specific brain cell type of AD-related genes and CBT-related genes both point to the serotonergic neurons, indicating the role of serotonergic neurons in genetic susceptibility and treatment response. In the human brain, serotonergic neurons are mainly located in the raphe nucleus and are implicated in fear, pain, and mood^39^. Serotonergic neurons are generally believed to play a central role in the pathogenesis and the pharmacotherapy of ADs^31^. For example, the activation of serotonergic neurons was shown to increase active coping in response to inescapable stress in rats and mice^40^. Serotonergic neurons are the unique resource of the neurotransmitter serotonin^41^, the dysfunction of which has been implicated in anxiety traits^42^ and antianxiety drugs^43,44^. Collectively, these findings highlight serotonergic neurons as a therapeutic target for ADs.

## Supplementary information

Supplementary Table 1-5

## Data Availability

Genes related to ADs are listed in Supplementary Table 1. Genes response to CBT of ADs are listed in Supplementary Table 2. Genes related to MDD and/or BIP are listed in Supplementary Table 3. Genes related to NEU are listed in Supplementary Table 4.

## Code availability

The code for cell-type specificity investigation is written in R-program and is released at GitHub (https://github.com/liuhankui/Anxiety/blob/main/script.r).
